# Ablation of *Bscl2*/seipin in hepatocytes does not cause metabolic dysfunction in congenital generalised lipodystrophy

**DOI:** 10.1242/dmm.042655

**Published:** 2020-01-13

**Authors:** George D. Mcilroy, Sharon E. Mitchell, Weiping Han, Mirela Delibegović, Justin J. Rochford

**Affiliations:** 1 The Rowett Institute, University of Aberdeen, Aberdeen AB25 2ZD, UK; 2 Aberdeen Cardiovascular and Diabetes Centre, University of Aberdeen, Aberdeen AB25 2ZD, UK; 3 Institute of Biological and Environmental Sciences, University of Aberdeen, Aberdeen AB24 2TZ, UK; 4 Laboratory of Metabolic Medicine, Singapore Bioimaging Consortium, Agency for Science, Technology and Research (A*STAR), Singapore 138667; 5 Institute of Medical Sciences, University of Aberdeen, Aberdeen AB25 2ZD, UK

**Keywords:** Lipodystrophy, BSCL2, CGL, Hepatocyte, AAV, CRISPR

## Abstract

Mutations affecting the *BSCL2* gene cause the most severe form of congenital generalised lipodystrophy (CGL). Affected individuals develop severe metabolic complications including diabetes and hepatic steatosis. *Bscl2*-deficient mice almost entirely reproduce the CGL phenotype. Adipose tissue-specific loss of *Bscl2* is also sufficient to cause early-onset generalised lipodystrophy in mice. However, these mice do not show severe metabolic dysfunction, even when challenged with a high-fat diet. Germline *Bscl2* loss in mice and *BSCL2* disruption in humans causes severe hepatic steatosis, and the encoded protein, seipin, has acknowledged roles in lipid accumulation. Given the critical role of the liver in glucose regulation, we speculated that intact hepatic *Bscl2* expression may protect adipose tissue-specific *Bscl2*-deficient mice from metabolic disease. To investigate this, we generated a novel mouse model in which *Bscl2* has been deleted in both adipose tissue and hepatocytes simultaneously using an adeno-associated viral vector. Despite achieving efficient disruption of *Bscl2* in the liver, hepatic lipid accumulation and metabolic homeostasis was unaffected in mice fed a high-fat diet for 4 weeks. We also investigated the consequences of *BSCL2* ablation in the human hepatocyte HepG2 cell line using CRISPR/Cas9 genome editing. No significant increases in lipid accumulation were observed in *BSCL2* knockout cell lines. Overall, we reveal that *Bscl2*/*BSCL2* does not appear to play a cell-autonomous role in the regulation of lipid accumulation in the liver. Loss of hepatic *BSCL2* is therefore unlikely to contribute significantly to the development of hepatic steatosis or metabolic dysfunction in this form of CGL.

## Abstract (precis)

**Editor's choice:** Hepatic *Bscl2* ablation in adipose tissue-specific *Bscl2* knockout mice does not cause metabolic dysfunction. CRISPR/Cas9 genome editing reveals that seipin does not play a cell-autonomous role in regulating hepatocyte lipid accumulation.

## INTRODUCTION

The principal function of adipose tissue is to safely store energy that is derived from the diet in the form of triglyceride (Rosen and Spiegelman, 2014). However, numerous distinct adipose tissue depots are known to exist, which are highly diverse and perform specialised functions dependent upon their location within the body (Zwick et al., 2018). Adipose tissue is also now well recognised as an endocrine organ, playing a vital role in the regulation of energy homeostasis and therefore overall metabolic health. This is clearly highlighted by conditions of adipose tissue dysfunction. In obesity, excessive levels of adiposity can lead to a lack of further lipid storage capacity. The overflow of lipids to non-adipose tissues can then lead to the development of metabolic complications such as type 2 diabetes, hepatic steatosis and cardiovascular disease. An inappropriate lack of functional adipose tissue can be equally detrimental to metabolic health, as is observed in genetic or acquired conditions of lipodystrophy (Hussain et al., 2019).

Congenital generalised lipodystrophy (CGL) type 2 (CGL2) is the most severe form of lipodystrophy observed in humans and results from mutations in the gene *BSCL2* (Magré et al., 2001). *BSCL2* encodes the protein seipin, which is localised to the endoplasmic reticulum (Windpassinger et al., 2004; Lundin et al., 2006). The loss of adipose tissue in CGL2 affects both metabolic and mechanical depots (Altay et al., 2017). Due to the inability to safely store lipids in adipocytes, patients with this form of lipodystrophy develop severe metabolic complications including type 2 diabetes, hepatic steatosis and hyperlipidaemia (Hussain et al., 2019). Therapeutic efforts have been made to treat the lipoatrophic and metabolic phenotypes that arise in this condition; however, these have been largely ineffective. For example, the PPARγ agonist rosiglitazone, which activates the master regulator of adipogenesis, failed to significantly increase fat mass stores in a single patient receiving this treatment for a year (Victoria et al., 2010). Alternatively, leptin-replacement therapy can be effective in reducing appetite, partially resolving hepatic steatosis and improving glycaemic regulation (Chong et al., 2010; Beltrand et al., 2007). However, leptin therapy is not widely available, does not resolve all features of CGL and prolonged use can lead to the development of leptin antibodies and progression to leptin resistance (Beltrand et al., 2010). Therefore, alternative treatment strategies are urgently required.

Studies have also been performed using *in vitro* and *in vivo* systems to model CGL2, in order to determine the molecular function and mechanisms associated with seipin deficiency. Inhibition of *Bscl2* in cell culture models of adipogenesis clearly indicate that seipin induction is an essential requirement for the formation of adipocytes (Payne et al., 2008; Chen et al., 2009). Four independent groups have also generated global *Bscl2* knockout mouse models (Cui et al., 2011; Chen et al., 2012; Prieur et al., 2013; Mcilroy et al., 2018b), all of which almost entirely recapitulate the metabolic phenotype observed in patients with this condition (Dollet et al., 2014). We recently investigated the consequences of adipose tissue-specific ablation of *Bscl2* and were surprised to discover that, despite the early development of generalised lipodystrophy, metabolic dysfunction failed to manifest in male mice (Mcilroy et al., 2018b). This was also observed in female mice, which only began to show subtle signs of metabolic complications when placed at thermoneutrality and challenged with a high-fat diet (Mcilroy et al., 2018a). These findings led us to hypothesise that loss of seipin in non-adipose tissues may contribute to the development of the full metabolic phenotype in seipin-deficient individuals. If true, non-adipose tissues could therefore become novel targets for therapeutic intervention.

Recent studies have raised the possibility that seipin may play an important, cell-autonomous role within the liver (Lounis et al., 2017; Li et al., 2019). This organ plays a crucial role in lipid and glucose homeostasis, both of which are perturbed in patients and mice lacking seipin. Therefore, the presence of hepatic *Bscl2* in our adipose tissue-specific model might provide protection from the development of metabolic disease. To investigate this, here we have additionally ablated *Bscl2* specifically in the hepatocytes of male and female adipose tissue-specific *Bscl2* knockout mice, using adeno-associated viral vectors. Furthermore, we have generated *BSCL2* knockout lines in the human hepatocyte HepG2 cell model using CRISPR/Cas9 genome editing. Overall, we find that the additional ablation of seipin from hepatocytes fails to cause development of metabolic dysfunction *in vivo* and does not lead to alterations in triglyceride accumulation *in vitro*. Our findings therefore indicate that seipin is unlikely to play a cell-autonomous role in the regulation of lipid accumulation in the liver, even in the background of lipodystrophy.

## RESULTS

### Characterisation of male adipose tissue-specific *Bscl2*-deficient mice at thermoneutrality

Adipose tissue-specific *Bscl2* knockout mice [Ad-B2^(−/−)^] surprisingly failed to develop the severe metabolic dysfunction observed in *Bscl2* null mice, despite a similar generalised lack of adipose tissue (Mcilroy et al., 2018b). Female Ad-B2^(−/−)^ mice also failed to develop metabolic dysfunction, even when housed at thermoneutrality (30°C). Indeed, only moderate alterations to metabolic homeostasis became apparent when female mice housed at thermoneutrality were challenged with a high-fat diet (Mcilroy et al., 2018a). We also previously showed that male Ad-B2^(−/−)^ mice have preserved brown adipose tissue (BAT) and display increased markers of thermogenesis in residual epidydimal white adipose tissue (EWAT) (Mcilroy et al., 2018b). To investigate whether thermogenesis was offering any protection from metabolic disease, we examined whether housing male Ad-B2^(−/−)^ mice at thermoneutrality would uncover the metabolic phenotype observed in global *Bscl2* knockout mouse models, which typically have used male mice (Cui et al., 2011; Chen et al., 2012; Prieur et al., 2013; Mcilroy et al., 2018b).

**Fig. 1.**
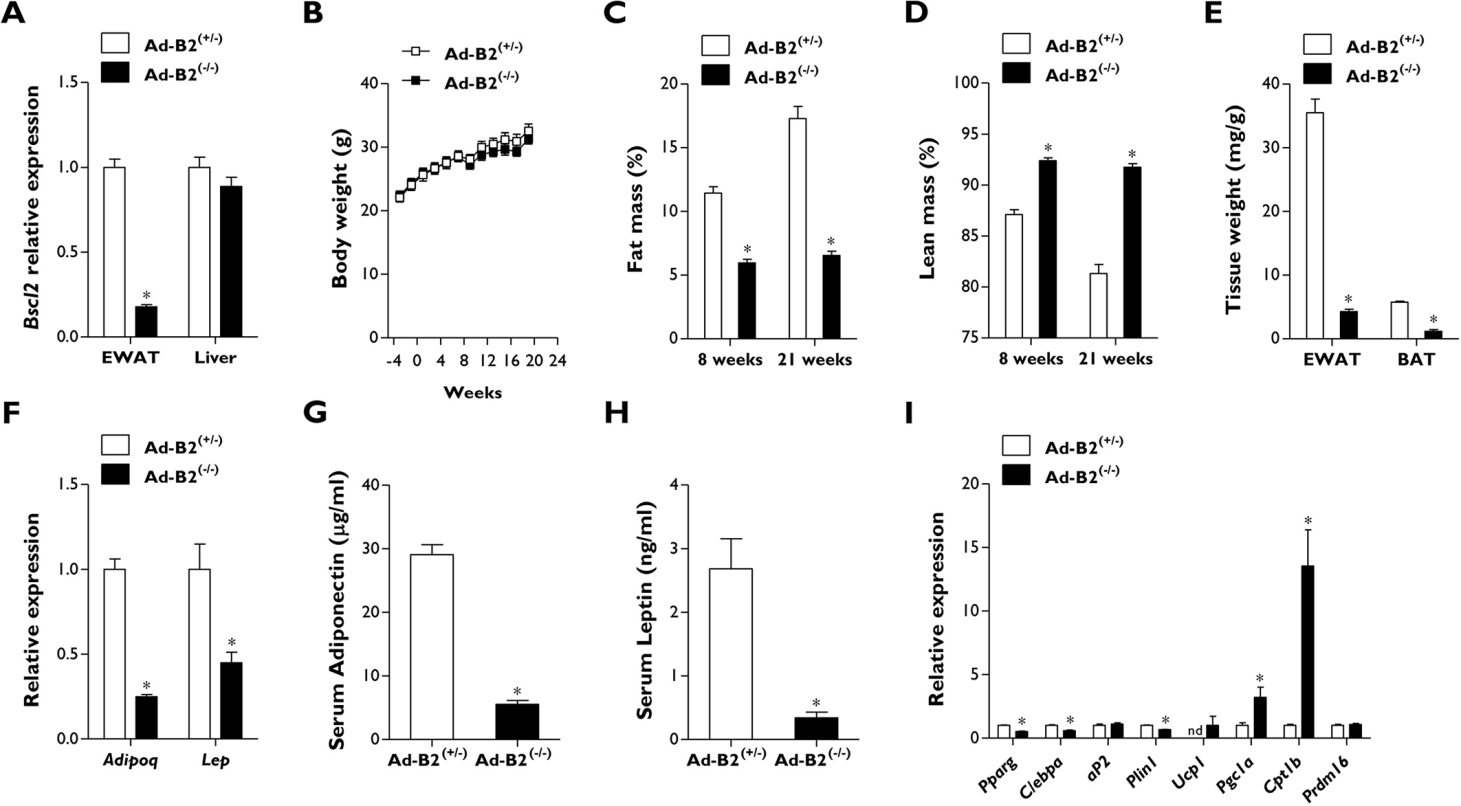
**Male Ad-B2^(−/−)^ mice housed at thermoneutrality are lipodystrophic.** (A) *Bscl2* mRNA levels in EWAT and liver of 32-week-old Ad-B2^(+/−)^ and Ad-B2^(−/−)^ mice housed at thermoneutrality (30°C) for 21 weeks. (B) Body weight progression of Ad-B2^(+/−)^ and Ad-B2^(−/−)^ mice as described in A. (C,D) Fat mass (C) and lean mass (D) levels assessed by DEXA and normalised to body weight in Ad-B2^(+/−)^ and Ad-B2^(−/−)^ mice after being housed at thermoneutrality for 8 and 21 weeks, respectively. (E) Tissue weights of EWAT and BAT of mice described in A normalised to body weight. (F) Relative gene expression levels of adiponectin and leptin from EWAT of mice described in A. (G,H) Serum adiponectin (G) and leptin (H) in mice described in A fasted for 5 h. (I) mRNA levels of white and brown adipocyte markers in EWAT of mice described in A. All data are biological replicates presented as the mean±s.e.m., *n*=6 mice for each group, *P<0.05 vs Ad-B2^(+/−)^. nd, not detected.

We placed a cohort of 11-week-old Ad-B2^(+/−)^ and Ad-B2^(−/−)^ male mice fed a chow diet at thermoneutrality (30°C) for a prolonged period of 21 weeks. We first confirmed the specificity of *Bscl2* ablation by quantitative PCR. *Bscl2* mRNA transcript levels were significantly decreased in EWAT in Ad-B2^(−/−)^ male mice compared to Ad-B2^(+/−)^ control mice; however, no significant differences in *Bscl2* gene expression levels were detected in the liver (Fig. 1A). No significant differences in body weight were observed between the two genotypes, both prior to and throughout the housing period at thermoneutrality (Fig. 1B). After 8 and 21 weeks at thermoneutrality, dual-energy X-ray absorptiometry (DEXA) analysis was performed. This revealed that Ad-B2^(−/−)^ male mice had significantly decreased fat mass (Fig. 1C) and significantly increased lean mass (Fig. 1D), as a percentage of body weight, compared to Ad-B2^(+/−)^ controls. Absolute fat mass was also significantly decreased at 8 and 21 weeks at thermoneutrality, and absolute lean mass showed a trend towards being increased; however, this was not significantly different at either time point (Fig. S1A,B). Interestingly, although bone mineral density was not significantly altered (Fig. S1C), both bone mineral content and bone area were found to be significantly increased in Ad-B2^(−/−)^ mice at both time points (Fig. S1D,E). Dissected tissue weight of EWAT was also significantly decreased, confirming the effect of *Bscl2* ablation on adipose tissue development (Fig. 1E). Housing Ad-B2^(−/−)^ mice at thermoneutrality also led to a severe and significant decrease in BAT tissue weight (Fig. 1E), compared to the relatively well-preserved BAT depot we observed in Ad-B2^(−/−)^ mice maintained at standard housing temperatures (Mcilroy et al., 2018b). Gene expression analysis of EWAT revealed significant decreases in *Adipoq* and *Lep* in Ad-B2^(−/−)^ mice compared to Ad-B2^(+/−)^ mice (Fig. 1F). Circulating serum levels of adiponectin and leptin in mice fasted for 5 h also showed significant decreases consistent with the changes in mRNA expression (Fig. 1G,H).

Both global *Bscl2* knockout mice and male Ad-B2^(−/−)^ mice have been shown to display elevated gene expression levels of thermogenic markers in residual EWAT depots (Chen et al., 2012; Mcilroy et al., 2018b). To determine whether this was also the case in male Ad-B2^(−/−)^ mice housed at thermoneutrality, markers of white and brown adipose tissue were examined. Levels of *Pparg*, *C/ebpa* and *Plin1* were significantly decreased in Ad-B2^(−/−)^ mice compared to controls; however, no significant changes were found for *aP2* (also known as *Fabp4*) (Fig. 1I). Curiously, significantly increased expression of markers of thermogenesis such a *Cpt1b* and *Pgc1a* (also known as *Ppargc1a*) were still apparent in residual EWAT in male Ad-B2^(−/−)^ mice. Additionally, expression of *Ucp1* was observed in Ad-B2^(−/−)^ mice; however, it was undetectable in heterozygous control animals. No significant changes were observed for *Prdm16* between genotypes (Fig. 1I).

### Male Ad-B2^(−/−)^ mice housed at thermoneutrality do not develop metabolic dysfunction

**Fig. 2.**
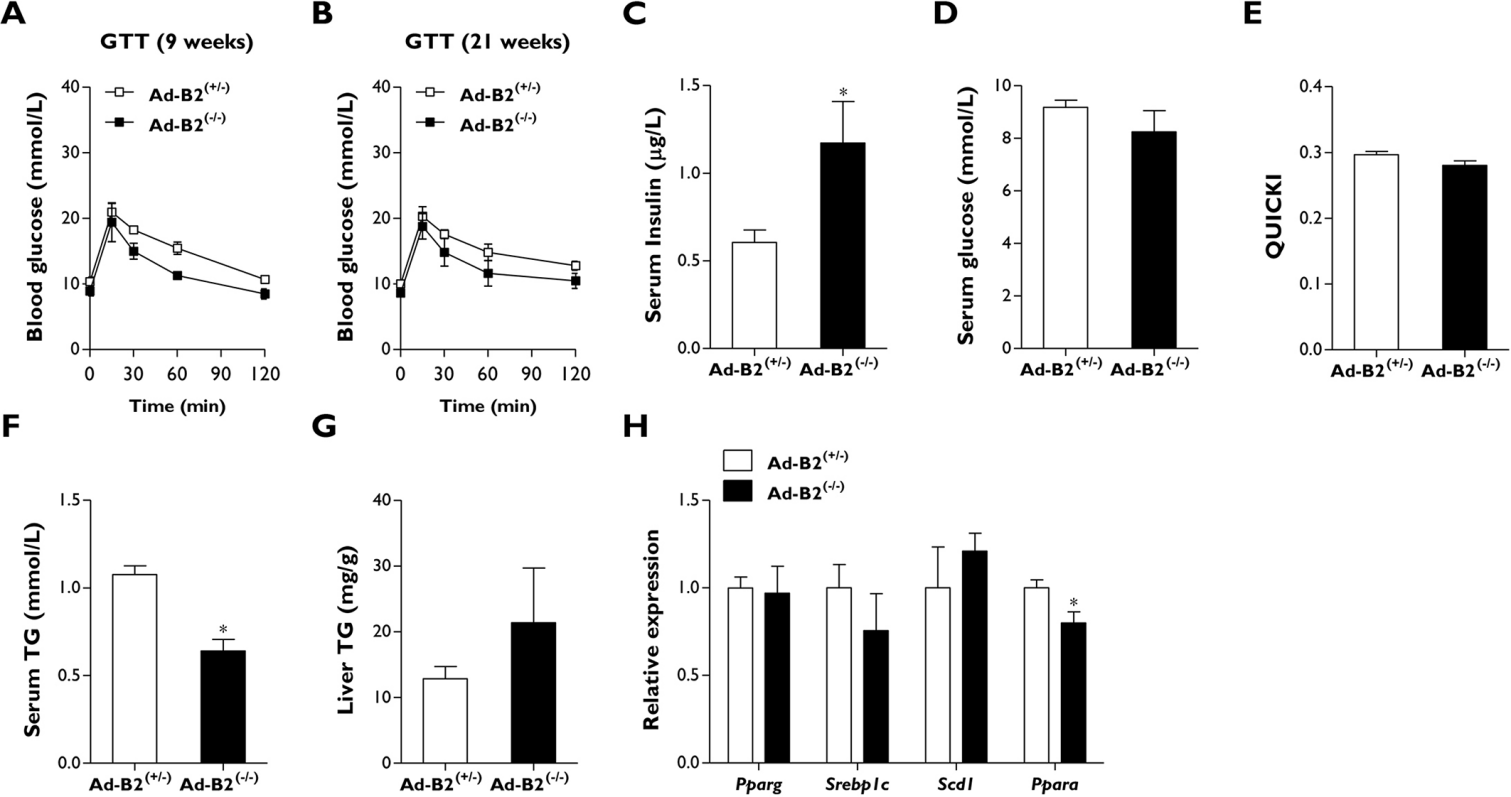
**Male Ad-B2^(−/−)^ mice housed at thermoneutrality are not glucose intolerant.** (A) Glucose tolerance tests (GTT) performed in Ad-B2^(+/−)^ and Ad-B2^(−/−)^ mice housed at thermoneutrality for 9 weeks. The glucose bolus was normalised to body weight. (B) Glucose tolerance tests performed in Ad-B2^(+/−)^ and Ad-B2^(−/−)^ mice housed at thermoneutrality for 21 weeks. The glucose bolus was normalised to lean mass. (C-F) Serum insulin (C), serum glucose (D), QUICKI (E) and serum triglyceride (TG) levels (F) in Ad-B2^(+/−)^ and Ad-B2^(−/−)^ male mice housed at thermoneutrality for 21 weeks and fasted for 5 h. (G) Liver TG levels normalised to tissue weight. (H) Relative gene expression levels of lipid-related markers in the liver of Ad-B2^(+/−)^ and Ad-B2^(−/−)^ male mice. All data are biological replicates presented as the mean±s.e.m., *n*=6 (Ad-B2^(+/−)^) and *n*=5 (Ad-B2^(−/−)^) mice for each group, *P<0.05 vs Ad-B2^(+/−)^.

Female Ad-B2^(−/−)^ mice housed at thermoneutrality and fed a chow diet failed to develop the metabolic disease observed in global *Bscl2* knockout mice (Mcilroy et al., 2018a). To determine whether this was also the case in Ad-B2^(−/−)^ male mice, we performed glucose tolerance tests after housing mice at thermoneutrality for 9 weeks. No significant differences were observed between genotypes when the injected glucose bolus was normalised to body weight (Fig. 2A). After housing mice at thermoneutrality for 21 weeks, glucose tolerance tests were repeated. Due to the observed differences in the percentage of lean mass between Ad-B2^(+/−)^ and Ad-B2^(−/−)^ mice (Fig. 1D), we injected glucose normalised to lean mass levels to determine whether this was altering glucose clearance in Ad-B2^(−/−)^ male mice. Even under these conditions we observed no significant alterations in glucose clearance between control and Ad-B2^(−/−)^ mice (Fig. 2B).

We next examined serum parameters to determine whether any alterations were apparent. These were measured in mice that had been housed at thermoneutrality for 21 weeks and had been fasted for 5 h. A significant increase in circulating insulin was observed in Ad-B2^(−/−)^ mice (Fig. 2C); however, serum glucose levels and quantitative insulin sensitivity check index (QUICKI) analysis results were not significantly different (Fig. 2D,E), indicating that male Ad-B2^(−/−)^ mice were not insulin resistant. Serum triglyceride levels were found to be significantly decreased in Ad-B2^(−/−)^ male mice (Fig. 2F), which is also observed in global *Bscl2* knockout mice when fasted for similar periods (Cui et al., 2011; Chen et al., 2012; Prieur et al., 2013). Liver triglyceride levels appeared to be slightly elevated in Ad-B2^(−/−)^ male mice compared to controls (Fig. 2G); however, this difference was not significant. Liver gene expression levels of *Pparg*, *Srebp1c* (also known as *Srebf1*) and *Scd1* were not significantly altered; however, a small but significant decrease in *Ppara* expression was detected in Ad-B2^(−/−)^ male mice (Fig. 2H).

Overall, these results revealed that despite housing lipodystrophic male Ad-B2^(−/−)^ mice at thermoneutrality for a prolonged period of time, this model fails to develop the severe metabolic dysfunction observed in global *Bscl2* knockout mouse models.

### Additional ablation of hepatic *Bscl2* in male and female Ad-B2^(−/−)^ mice

From the findings presented above along with previously published data (Mcilroy et al., 2018a,b), it appears that adipose tissue-specific deficiency of *Bscl2* is insufficient to reproduce the metabolic phenotype observed in global *Bscl2* knockout mice. Indeed, all other single tissue-specific *Bscl2* deficient mouse models generated to date have failed to report a metabolic phenotype (Chen et al., 2014; Zhou et al., 2014; Xu et al., 2019). As *BSCL2* is widely expressed in several tissues (Magré et al., 2001), we hypothesised that loss of *Bscl2* in non-adipose tissues may be necessary for severe metabolic dysfunction to develop in conditions of CGL2. We therefore decided to additionally ablate *Bscl2* in the liver of male and female Ad-B2^(−/−)^ mice using adeno-associated viral vectors, due to the critical role of this tissue in regulating glucose and lipid homeostasis.

**Fig. 3.**
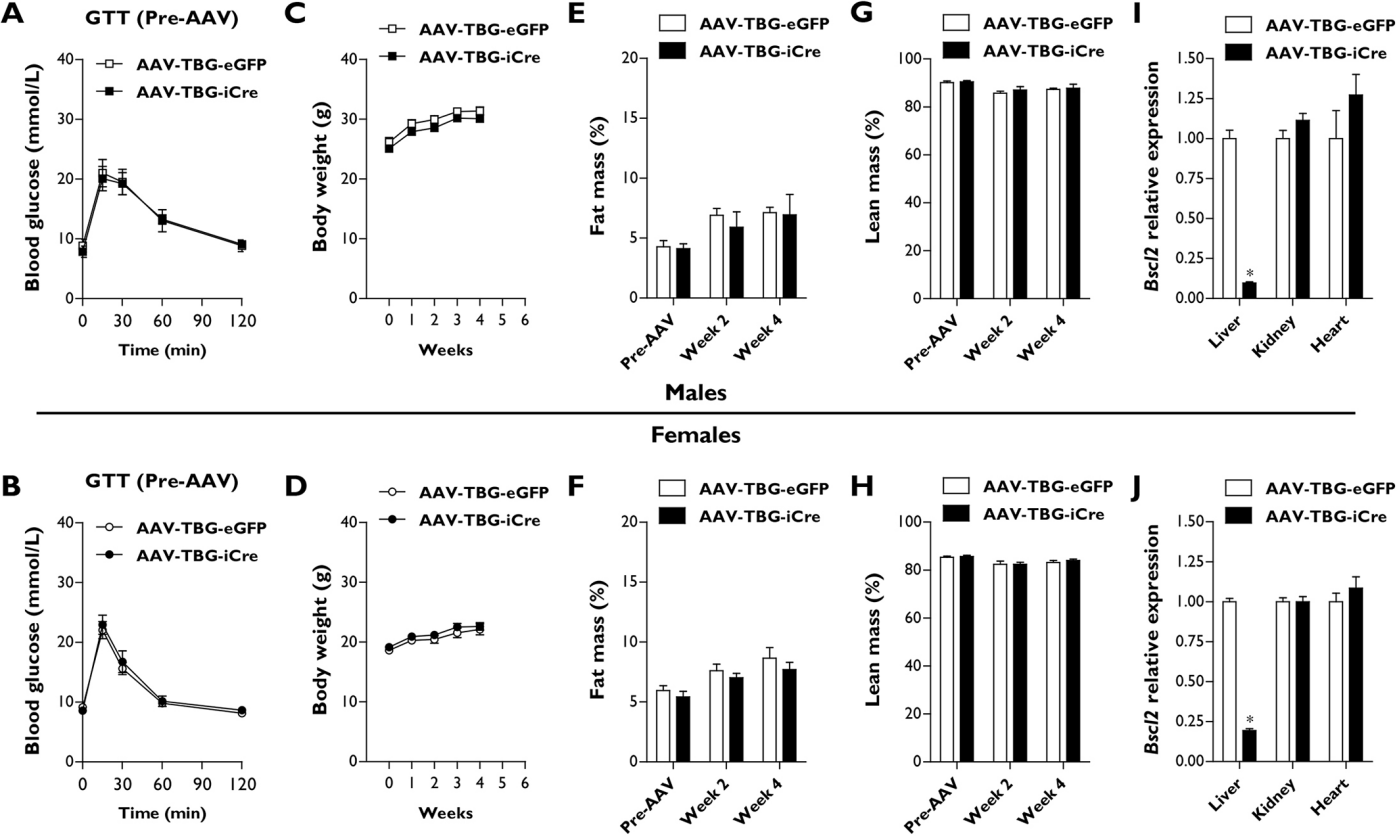
**Characterising male and female Ad-B2^(−/−)^ mice with AAV-mediated hepatic *Bscl2* ablation.** (A,B) Baseline (Pre-AAV) glucose tolerance tests with glucose bolus normalised to body weight in male (A) and female (B) Ad-B2^(−/−)^ mice. (C,D) Body weight progression of male (C) and female (D) Ad-B2^(−/−)^ mice injected with AAV-TBG-eGFP and AAV-TBG-iCre vectors and fed a high-fat diet for 4 weeks. (E-H) Fat mass (E,F) and lean mass (G,H) levels in male and female mice at baseline (Pre-AAV), 2 or 4 weeks after AAV-TBG-eGFP or AAV-TBG-iCre injection assessed by EchoMRI and normalised to body weight. (I,J) Relative *Bscl2* mRNA levels in liver, kidney and heart of male (I) and female (J) AAV-TBG-eGFP and AAV-TBG-iCre mice. All data are biological replicates presented as the mean±s.e.m., *n*=6 (eGFP) and *n*=5 (iCre) mice for males, *n*=7 (eGFP) and *n*=8 (iCre) mice for females, *P<0.05 vs AAV-TBG-eGFP.

To do this, we generated a cohort of 8- to 12-week-old male and female Ad-B2^(−/−)^ mice and examined basal (Pre-AAV) glucose tolerance (Fig. 3A,B), body weight (Fig. 3C,D), fat mass (Fig. 3E,F) and lean mass (Fig. 3G,H) levels. Mice were then randomised into two groups that were not significantly different for each parameter. Mice received intraperitoneal injections of 1.5×10^11^ genome copies of an adeno-associated virus–thyroxine-binding globulin promoter–eGFP (AAV-TBG-eGFP) control virus or adeno-associated virus–thyroxine-binding globulin promoter–Cre recombinase vector (AAV-TBG-iCre), in order to direct hepatocyte-specific recombination and deletion of *Bscl2*. Mice were then fed a high-fat diet (60% kcal from fat) and monitored for a period of 4 weeks at standard housing temperatures (21°C). No significant alterations to body weight (Fig. 3C,D), fat mass (Fig. 3E,F) or lean mass (Fig. 3G,H) were observed in either male or female Ad-B2^(−/−)^ mice injected with AAV-TBG-iCre compared to AAV-TBG-eGFP control animals during the 4-week monitoring period.

To confirm efficient and specific targeting of *Bscl2* by the AAV-TBG-iCre vector, we performed quantitative PCR analysis. Liver *Bscl2* gene expression levels were significantly decreased by ∼90% in male mice (Fig. 3I) and by ∼80% in female mice (Fig. 3J). However, no significant changes to *Bscl2* gene expression were detected in either the kidney or heart of male or female mice treated with AAV-TBG-iCre compared to AAV-TBG-eGFP controls (Fig. 3I,J). The data presented indicate that AAV-TBG-iCre targeting of *Bscl2* in Ad-B2^(−/−)^ male and female mice is highly efficient and specific; therefore, establishing a novel mouse model in which *Bscl2* has been ablated in adipose tissue and hepatocytes simultaneously. Importantly, this method of targeting does not lead to alterations to body weight, fat mass or lean mass compared to AAV-TBG-eGFP control animals.

### Loss of hepatic *Bscl2* in male and female Ad-B2^(−/−)^ mice does not cause metabolic dysfunction

**Fig. 4.**
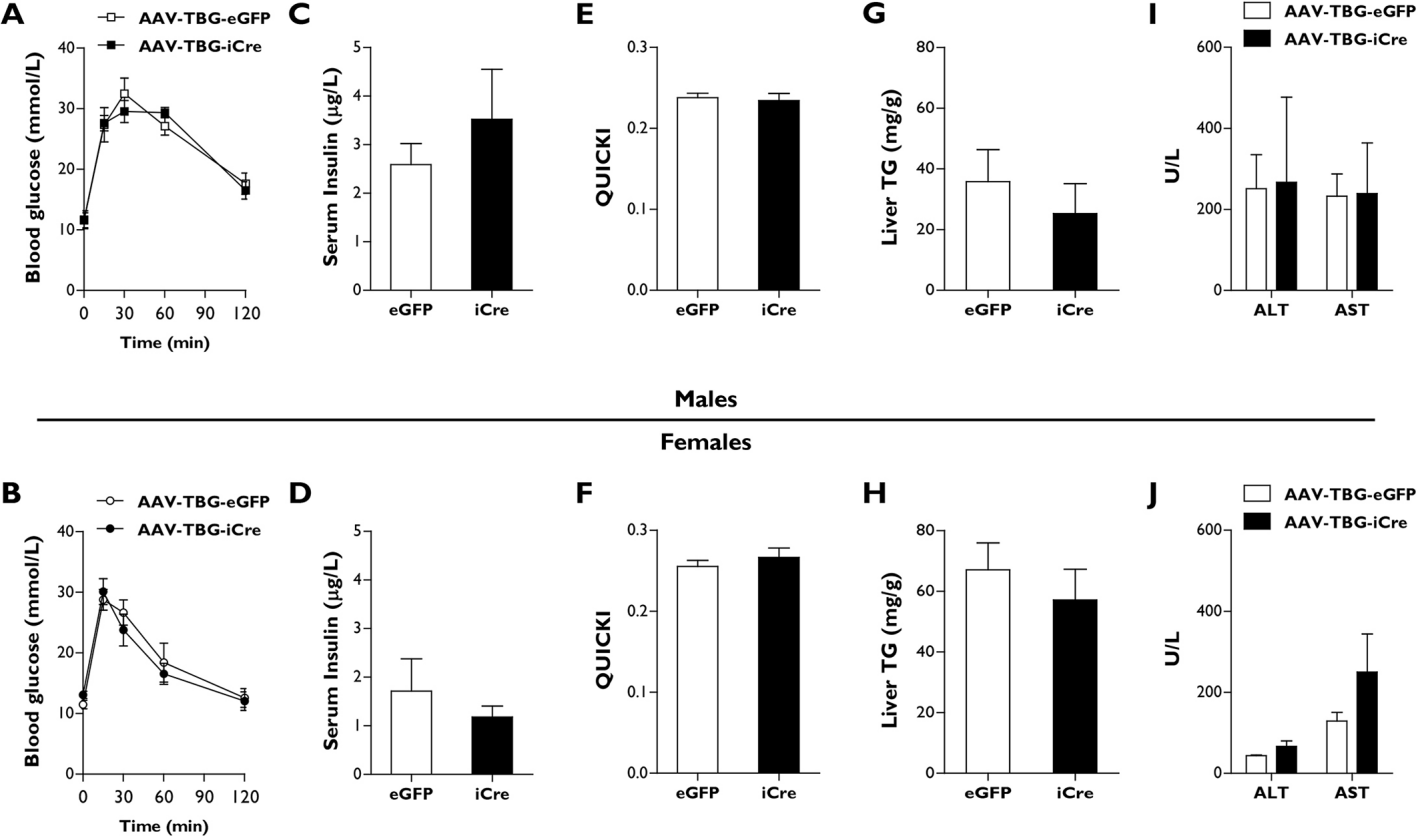
**Male and female AAV-TBG-iCre mice do not develop metabolic dysfunction.** (A,B) Glucose tolerance tests (Week 4) in which the glucose bolus was normalised to body weight in male (A) and female (B) AAV-TBG-eGFP and AAV-TBG-iCre mice. (C-J) Serum insulin (C,D), QUICKI analysis (E,F), liver TG levels (G,H) and serum AST and ALT levels in male (I) and female (J) AAV-TBG-eGFP and AAV-TBG-iCre mice fasted for 5 h. All data are biological replicates presented as the mean±s.e.m., *n*=6 (eGFP) and *n*=5 (iCre) mice for males, *n*=7 (eGFP) and *n*=8 (iCre) mice for females.

We next determined whether the additional loss of hepatic *Bscl2* would be sufficient to cause metabolic dysfunction in lipodystrophic Ad-B2^(−/−)^ mice. Glucose tolerance tests were performed 4 weeks after mice had been injected with AAV-TBG-iCre vectors and fed a high-fat diet. We found no significant differences in male or female AAV-TBG-iCre mice compared to controls in their ability to clear the injected glucose bolus (Fig. 4A,B). Similarly, when serum insulin levels (Fig. 4C,D) or serum triglyceride levels (Fig. S1F,G) were examined, no significant differences were observed. Additionally, QUICKI analysis revealed that the additional loss of hepatic *Bscl2* had failed to induce insulin resistance in male or female AAV-TBG-iCre mice compared to their AAV-TBG-eGFP injected littermate controls (Fig. 4E,F).

We next investigated whether the ablation of *Bscl2* in hepatocytes in the background of lipodystrophy would lead to the development of hepatic steatosis that is commonly observed in the global *Bscl2* knockout mouse models. No significant alterations to liver triglyceride levels were detectable in either male or female mice (Fig. 4G,H), despite the substantial decrease in *Bscl2* expression levels within this tissue (Fig. 3I,J). Additionally, we observed no significant alterations in hepatic gene expression levels of *Pparg*, *Srebp1c* or *Scd1* in male or female AAV-TBG-iCre mice (Fig. S1H,I). We also saw no significant changes in *Ppara* expression in female mice (Fig. S1I); however, once again, a small but significant decrease in *Ppara* expression was detected in Ad-B2^(−/−)^ male mice (Fig. S1H). To determine whether there was any indication of the development of liver damage in Ad-B2^(−/−)^ mice injected with AAV-TBG-iCre, we examined serum levels of alanine aminotransferase (ALT) and alanine transaminase (AST). In agreement with a lack of lipid accumulation in the liver and alterations to hepatic gene expression levels, we found no significant alterations to ALT or AST levels in male or female AAV-TBG-iCre mice compared to controls (Fig. 4I,J). Similarly, no significant differences in the AST/ALT ratio were detected (data not shown). Overall, our data indicate that loss of *Bscl2* in hepatocytes in the background of lipodystrophy fails to cause metabolic dysfunction and the severe hepatic steatosis observed in global *Bscl2* knockout mice. Therefore, it would appear that *Bscl2* loss in hepatocytes does not play a significant role in the development of hepatic steatosis or glucose intolerance in seipin-deficient states, even on a background of generalised lipodystrophy.

### Ablation of *BSCL2* in a human hepatocyte cell line does not alter lipid accumulation

Our *in vivo* findings are in keeping with previously published data in which liver-specific *Bscl2* knockout mice did not develop glucose intolerance or hepatic steatosis, even when challenged with a high-fat diet (Chen et al., 2014). However, recent studies have indicated that knockdown of *Bscl2*/*BSCL2* in both primary mouse hepatocytes and the human hepatocyte HepG2 cell line leads to alterations in lipid droplet morphology and triglyceride accumulation (Lounis et al., 2017). Additionally, a recent study has also shown that overexpression of *Bscl2* in mice may lead to reductions in hepatic triglyceride levels (Li et al., 2019). These findings therefore imply that *Bscl2* could indeed play a cell-autonomous role within the liver.

**Fig. 5.**
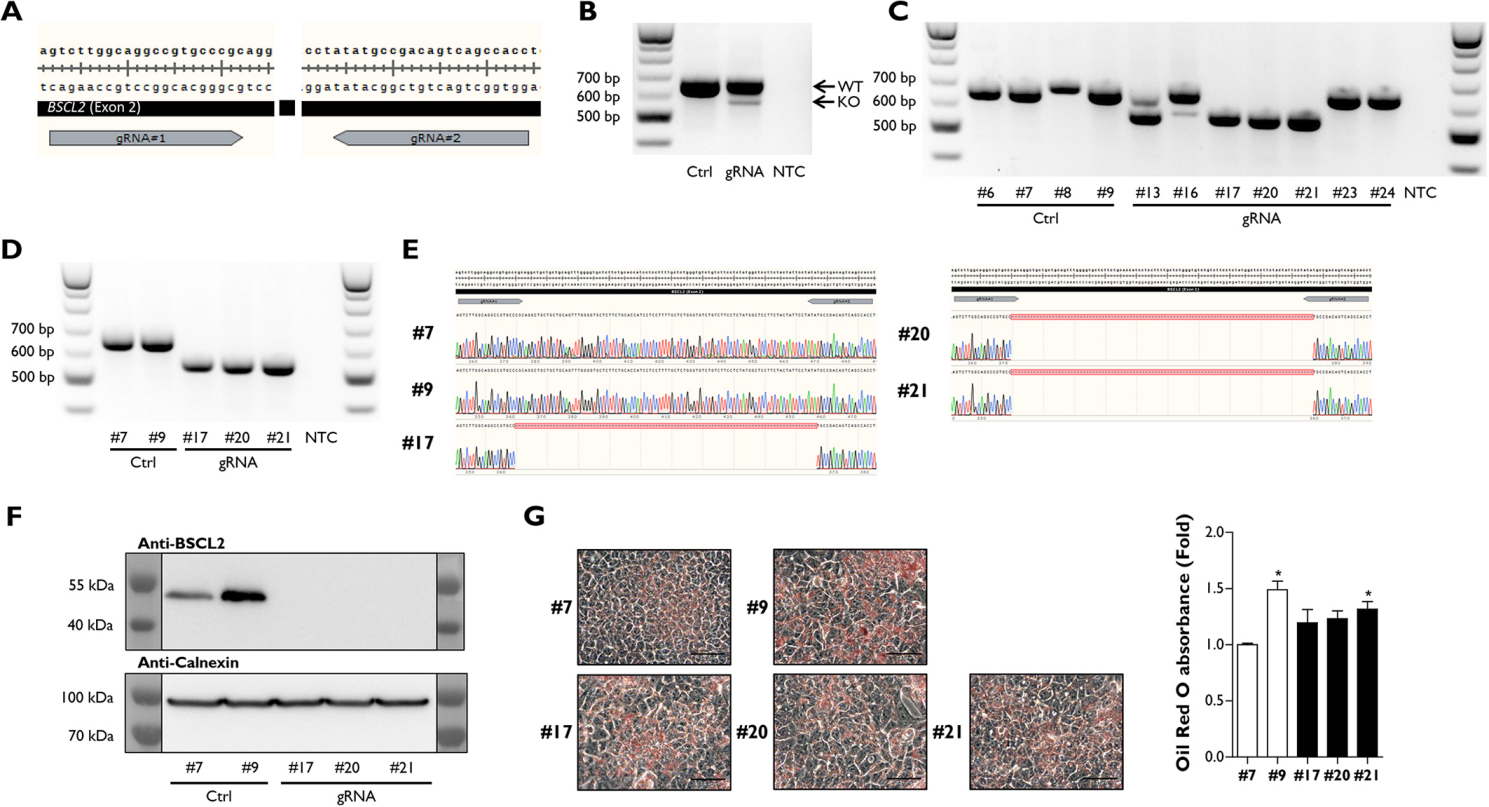
**Ablation of *BSCL2* in HepG2 cells does not alter lipid accumulation.** (A) Schematic representation of CRISPR/Cas9 gRNA editing sites in exon 2 of *BSCL2*. (B) PCR identification of *BSCL2* exon 2 deletion by CRISPR/Cas9 in a mixed population of HepG2 cells. KO, knockout; NTC, no template control; WT, wild type. (C) PCR identification of single-cell HepG2 colonies containing *BSCL2* exon 2 deletion isolated by serial dilution. (D,E) PCR analysis of clones selected for expansion (D) and DNA sequencing (E) of PCR products confirming *BSCL2* exon 2 deletion in HepG2 cells. (F) Western blot analysis confirming the ablation of BSCL2 protein in HepG2 single cell clones with *BSCL2* exon 2 deletion. (G) Representative images of Oil Red O-stained HepG2 cell clones (scale bars: 50 µm) and quantification of the eluted stain from biological replicates (*n*=8); data presented are from two independent experiments. Data are presented as the mean±s.e.m., *P<0.05 vs Ctrl #7.

In order to investigate this further, we used CRISPR/Cas9 genome editing to knock out the *BSCL2* gene in the human HepG2 hepatocyte cell line. To do this, we designed two guide RNA (gRNA) sequences to target exon 2 and create a 97-bp deletion in the *BSCL2* gene (Fig. 5A). HepG2 cells were electroporated with either the two gRNA sequences or a control plasmid (Ctrl), which was used to generate a pool of unedited HepG2 cells, which had otherwise been treated identically. We first analysed the Ctrl and gRNA-electroporated populations by PCR to determine whether the 97-bp deletion was present. A minor PCR product was detectable at the anticipated size (Fig. 5B). We next generated single-cell HepG2 populations from both the Ctrl and gRNA mixed populations by serial dilution. PCR analysis indicated that a mixture of wild-type (clones #23 and #24), heterozygous (clones #13 and #16) and knockout (clones #17, #20 and #21) cell lines had been isolated, whilst all Ctrl cell lines (clones #6, #7, #8 and #9) were wild type (Fig. 5C). Clones #7 and #9 from Ctrl transfections and clones #17, #20 and #21 from gRNA transfections were expanded, and PCR analysis was repeated to confirm the previous results (Fig. 5D) and obtain DNA sequencing analysis to assess successful genome editing by CRISPR/Cas9 (Fig. 5E). We next established that the 97-bp deletion had inactivated the *BSCL2* protein product seipin by western blot analysis. We observed that seipin was completely absent in all three gRNA-transfected single-cell clones in comparison to Ctrl-transfected clones, in which seipin was detectable at the appropriate molecular mass (Fig. 5F).

Having generated three individual cell clones deficient in *BSCL2*, we investigated whether loss of seipin in hepatocytes would lead to any alterations in lipid accumulation. We plated each isolated clone at similar cell densities and then determined lipid accumulation levels by Oil Red O staining. We observed no obvious visual differences in the amount of lipid accumulation between cell clones. When we extracted and quantified the Oil Red O stain from HepG2 clones, we did observe a significant difference between Ctrl #7 compared to Ctrl #9 and gRNA #21 (Fig. 5G). These small differences may result from natural variation in lipid storage when single-cell populations are isolated. However, our data clearly demonstrate that the complete ablation of *BSCL2* does not significantly increase lipid accumulation in hepatocytes. Overall, our *in vitro* findings agree with our observations and those of others *in vivo*, that loss of hepatic *Bscl2* does not appear to cause severe hepatic steatosis or metabolic dysfunction and is therefore unlikely to play a significant cell-autonomous role in the regulation of lipid accumulation within hepatocytes.

## DISCUSSION

Individuals with homozygous mutations affecting the *BSCL2* gene develop the most severe form of CGL (Magré et al., 2001). This disorder is characterised by the near complete absence of both metabolic and mechanical adipose tissue depots and the development of severe metabolic dysfunction (Altay et al., 2017; Hussain et al., 2019). Mouse models deficient in *Bscl2* almost entirely reproduce this phenotype (Cui et al., 2011; Chen et al., 2012; Prieur et al., 2013; Mcilroy et al., 2018b), providing a valuable *in vivo* tool to investigate and characterise this rare genetic disorder. To explore this condition further, mouse models examining the consequences of *Bscl2* deficiency using tissue-specific promoters have also been generated. Owing to the critical role of adipose tissue in energy homeostasis, it is perhaps not surprising that metabolic dysfunction was not reported when *Bscl2* was depleted specifically in the liver (Chen et al., 2014), brain (Zhou et al., 2014) or muscle (Xu et al., 2019). Unexpectedly, however, glucose intolerance and severe hepatic steatosis was not observed when we specifically targeted *Bscl2* in adipose tissue driven by Adipoq-Cre (Mcilroy et al., 2018b). Indeed, we have failed to observe these metabolic consequences, which are found in global *Bscl2* knockout mice, even after challenging female Ad-B2^(−/−)^ mice with thermoneutral housing conditions and feeding them a high-fat diet (Mcilroy et al., 2018a). We now also report that male Ad-B2^(−/−)^ mice housed at thermoneutrality also fail to develop metabolic dysfunction. This reveals that preserved brown adipose tissue thermogenesis and induction of thermogenic markers in residual epidydimal white adipose tissue is unlikely to explain the lack of glucose intolerance in these mice, despite generalised lipodystrophy.

These findings led us to hypothesise that the development of metabolic dysfunction in CGL2 may require the additional loss of *Bscl2* in non-adipose tissues. The liver appeared to be the most likely tissue to buffer the effects of adipose loss for several reasons. First, this organ plays a crucial role in glucose and lipid homeostasis. Second, the development of severe hepatic steatosis is a hallmark observed in patients and mice that are deficient in seipin (Dollet et al., 2014; Akinci et al., 2016; Hussain et al., 2019). Third, recent studies examining *Bscl2*/*BSCL2* loss of function or its overexpression in hepatocytes have indicated that seipin may play an important cell-autonomous role in the liver (Lounis et al., 2017; Li et al., 2019). To investigate this, we have used adeno-associated viral vectors to generate a novel mouse model, in which *Bscl2* has been specifically ablated in both adipose tissue and hepatocytes simultaneously. Despite significant decreases in liver *Bscl2* expression levels in male and female Ad-B2^(−/−)^ mice (∼90% and ∼80%, respectively), we observed no significant alterations to multiple *in vivo* metabolic parameters compared to control mice, even when challenged with a high-fat diet for 4 weeks. Our findings are in keeping with those of Chen et al. (2014), where *Bscl2* liver-specific knockout mice failed to develop a metabolic phenotype when fed either a chow or high-fat diet. Although we speculated that the lack of lipodystrophy in that model may have protected against metabolic dysfunction, our data now provide strong additional evidence that *Bscl2* is unlikely to play a cell-autonomous role in the liver, even in the presence of lipodystrophy. We are unaware of any other studies that have combined a genetically driven tissue-specific knockout mouse model with AAV-Cre infection to selectively ablate a gene of interest in an additional tissue. Indeed, we believe that there may only be one other published study in which genetically driven Cre recombination has been used to specifically target a single gene in two tissue types (adipocytes and endothelial cells) at the same time (Crewe et al., 2018). The novelty and ease of our targeting method will therefore be broadly applicable to studies aiming to investigate the crosstalk between tissues and the individual contributions of each.

*In vitro* investigations identifying a possible cell-autonomous role for *Bscl2*/*BSCL2* in hepatocytes achieved approximately a 50% reduction in seipin protein levels by small interfering RNA (Lounis et al., 2017). To investigate the complete absence of *BSCL2* in hepatocytes, we generated HepG2 *BSCL2* knockout cell lines using CRISPR/Cas9 genome editing. Despite the complete absence of seipin protein, we found no significant increase in the amount of triglyceride present in knockout cells. Although we have not specifically investigated whether alterations to lipid droplet size or morphology are present, our *in vitro* findings are consistent with our *in vivo* studies. Together, these reveal that hepatic deficiency of seipin does not appear to be responsible for significant increases in hepatocyte lipid content or the severe hepatic steatosis that is observed in global *Bscl2* knockout mouse models or patients suffering from CGL2.

If the additional loss of seipin in hepatocytes also fails to induce a metabolic phenotype, then this raises important questions about the differences between the global and conditional *Bscl2* knockout models. Our *Bscl2* adipose tissue-specific knockout mice appear to be similarly lipodystrophic compared to seipin knockout mice. However, the infrapatellar fat pad was found to be well preserved in Ad-B2^(−/−)^ mice, yet completely absent in SKO mice (Mcilroy et al., 2018b). Caudal marrow adipose tissue located in the vertebrae of the tail was also present in Ad-B2^(−/−)^ mice and is likely to be absent in SKO mice, although this has yet to be examined. Therefore, our previous findings (Mcilroy et al., 2018a,b), along with those presented in this paper, would imply that these and other small additional residual adipose tissue depots that remain may be sufficient to prevent the development of metabolic dysfunction associated with lipodystrophy in CGL2. Consistent with this notion, adipose tissue transplantation studies have also been shown to successfully alleviate the metabolic dysfunction observed in SKO mice (Liu et al., 2018; Wang et al., 2019). Thus, our findings may indicate that rescuing only very small quantities of endogenous adipose tissue could be therapeutically beneficial as a treatment for affected individuals. Nonetheless, if this is the case, it seems remarkable that these small, preserved depots offer sufficient protection from metabolic disease in Ad-B2^(−/−)^ mice in the face of high-fat feeding and thermoneutrality, when control mice substantially expand their adipose depots.

Intriguingly, a recent study has shown that rescuing adipose tissue in CGL2 could be a realistic possibility. Genetic loss of the rate limiting enzyme of lipolysis, adipose triglyceride lipase (Atgl; also known as Pnpla2), appeared to effectively rescue adipose tissue development and prevent metabolic complications in SKO mice (Zhou et al., 2019). The authors propose that Atgl is a direct downstream target of *Bscl2*, where even heterozygous deletion of *Atgl* seems to partially restore sufficient levels of adiposity to relieve metabolic complications. This appears to occur by preventing the increased rates of lipolysis from adipose tissue caused by uncontrolled cAMP/PKA activation, as previously identified due to *Bscl2* deficiency (Chen et al., 2012). Aside from the inherent challenges in achieving this, genetic manipulation of ATGL in CGL2 would risk ectopic lipid accumulation in the heart, which led to cardiac dysfunction and premature death of Atgl null mice *in vivo* (Haemmerle et al., 2006). This would seem particularly the case in CGL2 patients who typically display cardiomyopathy (Lupsa et al., 2010). However, pharmacological inhibition of Atgl, using the inhibitor Atglistatin, does not appear to cause the severe cardiac steatosis or cardiomyopathy observed in genetic models of ATGL disruption (Schweiger et al., 2017). It would be interesting to determine whether Atglistatin can restore adipose tissue mass in mature SKO mice. This seems unlikely if the lack of seipin leads to stalling and then failure of adipogenesis as has been proposed, which would result in a lack of nascent adipocytes on which the Atglistatin could act (Payne et al., 2008; Chen et al., 2009, 2012). Regardless of this, Zhou et al. (2019) have shown that adipose tissue restoration is possible in CGL. Pagac et al. (2016) also suggested that rescue of seipin-deficient pre-adipocytes may be possible through pharmacological inhibition of glycerol-3-phosphate acyltransferase 3 (Gpat3). However, the effects observed *in vitro* were modest and the ability of Gpat3 inhibition to rescue adipose tissue mass due to *Bscl2* deficiency has not yet been examined *in vivo*. Future studies investigating alternative pharmacological and gene therapeutic approaches are therefore warranted. If successful, these could prevent severe metabolic dysfunction in numerous forms of lipodystrophy and metabolic complications that arise in other forms of adipose tissue dysfunction, including conditions of obesity.

## MATERIALS AND METHODS

### Animal studies

*Bscl2* floxed mice (B2^(fl/fl)^) were generated as previously described (Mcilroy et al., 2018b). To generate adipocyte-specific seipin knockout mice (Ad-B2^(−/−)^), B2^(fl/fl)^ mice were crossed with heterozygous *Bscl2* floxed mice also carrying Cre recombinase driven by the *Adipoq* promoter (Ad-B2^(+/−)^). Adiponectin-Cre mice were generously provided by Dr Evan Rosen, Beth Israel Deaconess Medical Centre, Harvard Medical School, Boston, MA, USA. Animal procedures conducted on Ad-B2^(+/−)^ and Ad-B2^(−/−)^ mice were approved by the University of Aberdeen Ethics Review Board and performed under project licenses (PPL: P94B395EO and PFAD33FA2) approved by the UK Home Office. Calculations were performed to estimate mouse sample size (*n*) required to ensure adequate power to detect an effect. For studies performed at thermoneutrality, 11-week-old group-housed male mice were placed at 30°C for 21 weeks and exposed to a 12-h/12-h light-dark period. Mice were always given *ad libitum* access to water and a standard rodent chow diet [CRM (P) 801722, Special Diets Services] unless otherwise stated. Male Ad-B2^(+/−)^ mice that were littermates to Ad-B2^(−/−)^ male mice were used as controls. AAV-TBG-iCre experiments were performed at standard housing temperatures (21°C) using male and female Ad-B2^(−/−)^ mice, which were group housed by sex and exposed to a 12-h/12-h light-dark period. Tissues were rapidly dissected post-mortem, frozen in liquid nitrogen then stored at −70°C.

### Metabolic studies

Fat and lean mass levels were measured in Ad-B2^(+/−)^ and Ad-B2^(−/−)^ mice by DEXA (Lunar PIXImus) after being housed at thermoneutrality for 8 and 21 weeks. For AAV-TBG-iCre experiments, fat and lean mass levels were measured in 8- to 12-week-old Ad-B2^(−/−)^ male and female mice fed a chow diet and kept at standard housing temperatures using the EchoMRI™-500 body composition analyser (Zinsser Analytic GmbH) and then at 2 and 4 weeks after being fed a high-fat diet [60% kcal from fat (D12492), Research Diets]. Prior to glucose tolerance tests, mice were placed in clean cages and food was withheld for 5 h. Basal glucose readings (0 min) were determined by glucometer readings (AlphaTrak^®^ II, Zoetisus) from tail punctures. Mice were then given a 2 mg/g d-glucose (Sigma-Aldrich) bolus by intraperitoneal injection. Blood glucose levels were monitored at 15, 30, 60 and 120 min. Mice had *ad libitum* access to water throughout.

### AAV-TBG-iCre vector delivery

Prior to AAV vector injection, 8- to 12-week-old male and female Ad-B2^(−/−)^ mice fed a chow diet were randomised into two groups after basal body weights, glucose tolerance, fat and lean mass levels had been determined. Ad-B2^(−/−)^ mice were then injected with 1.5×10^11^ genome copies of AAV-TBG-iCre (Vector Biolabs, #VB1724) or AAV-TBG-eGFP (#VB1743) control vector via the intraperitoneal route (Ballantyne et al., 2016; Mehta et al., 2017). Ad-B2^(−/−)^ mice were then fed a high-fat diet [60% kcal from fat (D12492), Research Diets] for 4 weeks at standard housing temperatures. All mice had *ad libitum* access to food and water unless otherwise stated.

### Gene expression

Total RNA was extracted from frozen tissues using an RNeasy mini kit (Qiagen) following the manufacturer’s protocol. Equal quantities of RNA were DNase I treated (Sigma-Aldrich) then reverse transcribed with M-MLV reverse transcriptase, 5× reaction buffer, deoxynucleoside triphosphates and random primers (Promega). Real-time quantitative PCR was performed on a CFX384 Touch™ Real-Time PCR Detection System (Bio-Rad). No template controls and no reverse transcriptase controls were performed for every gene analysed. The geometric mean of three stable reference genes (*Nono*, *Ywhaz* and *Hprt*) was used for normalisation.

### Serum analysis

Blood was collected from 32-week-old chow-fed mice (thermoneutrality) or 12- to 16-week-old high-fat diet-fed mice (standard housing temperatures) fasted for 5 h by cardiac puncture. Blood was collected and inverted in SST™ amber tubes (BD Microtainer^®^) and incubated at room temperature for 30 min. Samples were then centrifuged at 12,000 ***g*** for 10 min and the separated serum collected. Insulin, adiponectin, leptin, AST and ALT analysis was performed at the Core Biochemical Assay Laboratory (Cambridge, UK). Glucose levels were determined using a Glucose Colorimetric Assay Kit (Cayman Chemical), following the manufacturer's protocol provided. Serum triglyceride levels were determined using a Triglyceride Liquid Assay (Sentinel Diagnostics), following the manufacturer's instructions. QUICKI was calculated from fasting glucose (mg/dl) and insulin (µU/ml) values as previously described (Katz et al., 2000). QUICKI=1/[log(I_0_)+log(G_0_)], where I_0_ is fasting insulin and G_0_ is fasting glucose. QUICKI is a dimensionless index without units.

### Liver triglyceride assay

Frozen liver tissue samples were weighed and then homogenised in 1 ml of PBS. Samples were kept on ice at all times. Liver lysates were centrifuged at 12,000 ***g*** for 10 min at 4°C. The supernatant was collected and triglyceride levels were determined using a Triglyceride Liquid Assay (Sentinel Diagnostics), following the manufacturer's instructions. Triglyceride levels were then normalised to individual tissue weights.

### Generation of *BSCL2* KO HepG2 cell lines by CRISPR/Cas9

HepG2 cells (Agouni et al., 2011) were cultured in Dulbecco's modified Eagle medium containing low glucose (1 g/l) and supplemented with 10% fetal bovine serum, 2% glutamine and 1% sodium pyruvate. Cells were maintained in a humidified incubator at 37°C with 5% CO_2_, HepG2 cells have not recently been authenticated or tested for contamination. Two gRNA sequences were identified using the online CRISPR guide tool software (http://crispr.mit.edu/) to target exon 2 of *BSCL2* and ligated into pX330 (gRNA #1: 5′-TCTTGGCAGGCCGTGCCCGC-3′) and pX459 (gRNA #2: 5′-GTGGCTGACTGTCGGCATAT-3′) plasmids (#42230 and #62988, respectively; Addgene). HepG2 cells (1×10^6^) were electroporated with 5 µg control (Ctrl) or gRNA plasmids using a Cell Line Nucleofector Kit V (Lonza, VCA-1003) and Nucleofector 2b Device (Lonza) using program T-28.

### Western blot analysis

HepG2 cell monolayers were scraped in RIPA lysis buffer containing cOmplete protease inhibitor cocktail (Roche). Protein concentrations were determined by BCA assay (Thermo Fisher Scientific). SDS-PAGE was performed using equal quantities of protein, which were transferred to polyvinylidene fluoride membrane using standard protocols. Primary antibodies used at 1:1000 dilution included anti-BSCL2 (#23846, Cell Signaling Technology) and anti-calnexin (ab75801, Abcam). Anti-rabbit horseradish peroxidase-conjugated secondary antibody was used at 1:5000 dilution (#7074, Cell Signaling Technology) and was visualized using enhanced chemiluminescence (ECL substrate, Illuminata).

### Statistical analyses

All data are presented as the mean±s.e.m. and were analysed by an unpaired two-tailed Student's *t*-test, one-way analysis of variance (ANOVA) with Tukey post hoc test or two-way repeated measures ANOVA with Bonferroni post hoc test as appropriate using GraphPad Prism. *P*<0.05 was considered statistically significant.

## Supplementary Material

Supplementary information
