# Supplementary material for: Ablation of Bscl2/seipin in hepatocytes does not cause metabolic dysfunction in congenital generalised lipodystrophy
Source: Dis Model Mech. 2020 Jan 13;13(1):dmm042655. doi: 10.1242/dmm.042655 (PMC6994952; doi:10.1242/dmm.042655)
Supplement: Supplementary information [file dmm-13-042655-s1.pdf]

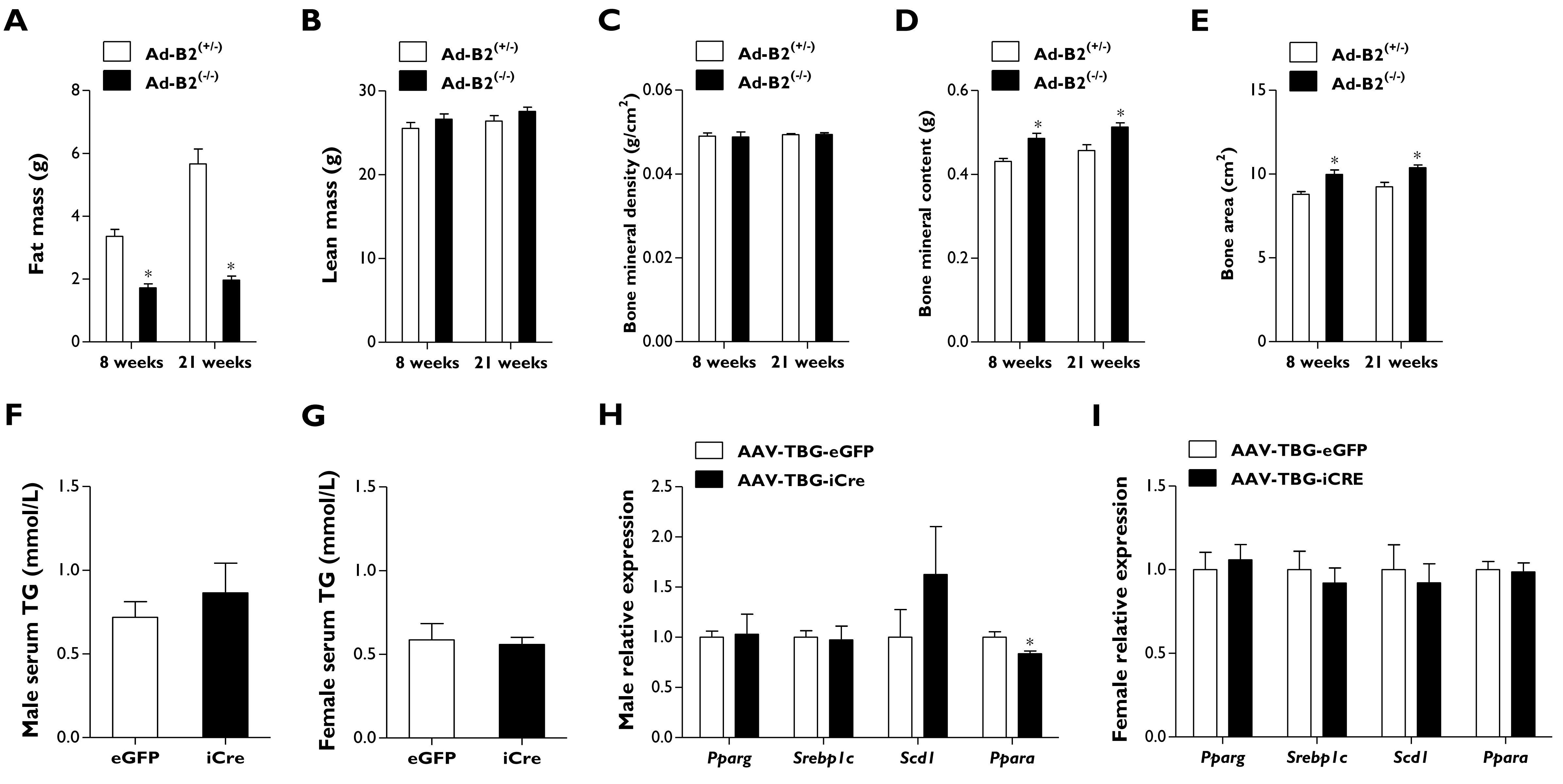

Fig S1

Figure S1. Absolute fat mass (**A**) and lean mass (**B**) levels, bone mineral density (**C**), bone mineral content (**D**) and bone area (**E**) assessed by DEXA in Ad-B2$^{(+/-)}$ and Ad-B2$^{(-/-)}$ male mice after being housed at thermoneutrality for eight and twenty-one weeks, n = 6 mice per group. Serum TG levels in male (**F**) and female (**G**) AAV-TBG-eGFP and AAV-TBG-iCre mice fasted for five hours. Relative gene expression levels of lipid related markers in the liver of male (**H**) and female (**I**) AAV-TBG-eGFP and AAV-TBG-iCre mice, n = 6 (eGFP) and 5 (iCre) mice for males, n = 7 (eGFP) and 8 (iCre) mice for females. All data are biological replicates presented as the mean ± SEM, *p<0.05 vs Ad-B2$^{(+/-)}$ or AAV-TBG-eGFP.

Disease Models & Mechanisms • Supplementary information
